# Orthognathic-Like Orthodontics: Management of Skeletal Class II Malocclusion in an Adult Patient

**DOI:** 10.7759/cureus.69628

**Published:** 2024-09-18

**Authors:** Dhruv Ahuja, Puneet Batra, Ashith MV, Ashish K Singh

**Affiliations:** 1 Orthodontics and Dentofacial Orthopedics, Manav Rachna Dental College, Manav Rachna International Institute of Research and Studies, Faridabad, IND; 2 Orthodontics and Dentofacial Orthopedics, Manipal College of Dental Sciences Mangalore, Manipal Academy of Higher Education, Mangalore, IND

**Keywords:** adult orthodontics, camouflage treatment, class ii malocclusion, maxillary molar distalization, miniscrew implants

## Abstract

Advances in orthodontic treatment, particularly with the use of temporary anchorage devices (TADs), have significantly improved outcomes for adult patients with skeletal Class II malocclusion. Traditionally reliant on orthognathic surgery, these malocclusions can now benefit from non-surgical options like maxillary molar distalization. Bone screws offer superior anchorage compared to conventional methods, enabling precise tooth movement without undesirable side effects.

This case report explores the efficacy of TAD-assisted maxillary molar distalization in an adult patient while focusing on a 50-year-old female who sought orthodontic treatment for Class II malocclusion, characterized by deep bite and increased overjet. Treatment involved the use of an infra-zygomatic crest (IZC) bone screw for maxillary molar distalization as a camouflage strategy to achieve optimal dento-skeletal and soft tissue profile improvements. The case also discusses the key considerations and benefits of utilizing bone screws in adult orthodontic treatment, particularly in minimizing reciprocal effects and reducing dependence on patient compliance.

## Introduction

Class II malocclusion affects 19.63% of adults, with mandibular underdevelopment occurring more often than maxillary prognathism [1]. Surgical intervention or orthodontic camouflage are the primary treatment options for adults having skeletal Class II malocclusion. Studies have shown that increasing vertical dimension through surgery or tooth movement in patients with a horizontal facial pattern with dental crowding is often unstable over time. Therefore, for adults with minimal skeletal discrepancies, orthodontic treatment that maintains the existing vertical dimension and extraction may be a suitable approach for achieving long-lasting results [2,3].

To address crowding, dental protrusion, and misaligned bite in adult patients with skeletal Class II, extraction of premolars is often done as part of camouflage treatment [4,5]. Alternatively, space can be created through interproximal stripping and distalizing the upper molars posteriorly. Traditional methods for moving these molars include using headgear, pendulum appliances, or distalizing springs [6]. To circumvent the challenges posed by patient non-compliance in orthodontic treatment, Kanomi introduced temporary anchorage devices (TADs) in the form of miniscrews implanted between the roots of posterior teeth [7]. This innovative approach aimed to facilitate the retraction of anterior teeth. These initially placed miniscrews were categorized as interradicular. However, subsequent research unveiled significant drawbacks associated with interradicular miniscrews, including a high failure rate, obstruction of tooth movement pathways, and encroachment on the roots of neighboring teeth. Specifically for maxillary distalization, the limitations of miniscrews would likely exacerbate due to the increased forces and complex mechanics involved in moving the entire maxilla posteriorly [8].

In recent years, mini-screws and mini-plates have become popular for correcting Class II malocclusion [9,10,11]. Skeletal anchorage provides an effective method for maxillary distalization in adult patients with Class II malocclusion, overcoming challenges related to patient cooperation [12]. In contrast to interradicular miniscrews, bone screws implanted in the infra zygomatic crest (IZC) region of the maxilla have emerged as a promising alternative. This placement allows for unimpeded tooth movement posteriorly as the miniscrews are positioned outside the root structure. The versatility of IZC miniscrews extends to various orthodontic treatments, including comprehensive maxillary distalization, correction of severe crowding, and asymmetry management. However, careful patient selection is crucial when employing IZC bone screws for achieving total maxillary dentition distalization in Class II patients [8].

This case report aims to assess the effectiveness of bone screws in distalization of the total maxillary arch in adult patients to achieve optimum dento-skeletal and soft tissue relation.

## Case presentation

**Figure 1:**
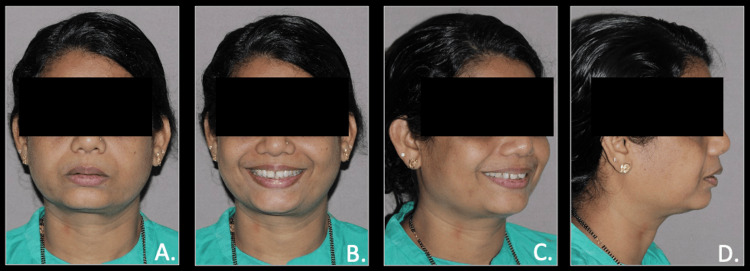
Pre-treatment extra-oral photographs A: frontal view; B: frontal smiling view; C: oblique smiling view; D: profile view

**Figure 2:**
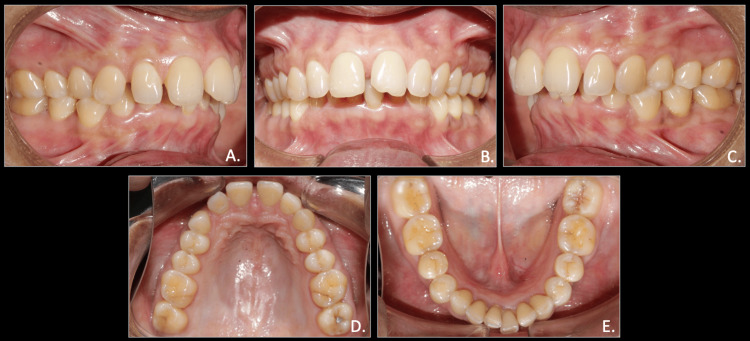
Pre-treatment intra-oral photographs A: right occlusion; B: anterior in occlusion; C: left occlusion; D: maxillary arch; E: mandibular arch

A 50-year-old woman sought dental care, expressing concerns about forwardly placed upper teeth and backwardly placed lower jaw. She reported previous health conditions of hypertension for more than five years and had undergone dental treatment of extraction with respect to both upper third molars and lower left third molar. The pre-treatment extra-oral photographs showed a convex facial profile with posterior facial divergence, protrusion of upper lips, a deep mentolabial sulcus, and potentially incompetent lips with retrognathic mandible. Intra-oral examination revealed Class II molar and canine relationships on the right and left sides, characterized by overcrowding in the lower arch, spacing in the upper arch, and a pronounced overbite and overjet (Figures 1, 2).

**Figure 3:**
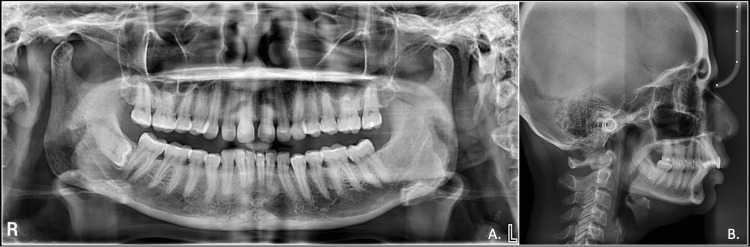
Pre-treatment radiographs A: OPG; B: lateral cephalogram OPG: orthopantomogram

Cephalometric evaluation revealed a skeletal Class II pattern and a decreased angle of the lower jaw and horizontal facial pattern. The upper and lower incisors were slightly proclined, and the upper lip was protrusive while the lower lip was retrusive from the esthetic line (E-line). The orthopantomogram revealed third molars in the maxilla and lower left quadrant were missing, while the lower right third molar was horizontally impacted. Overall, periodontal state was generally good with mild horizontal bone loss (Figure 3). 

**Figure 4:**
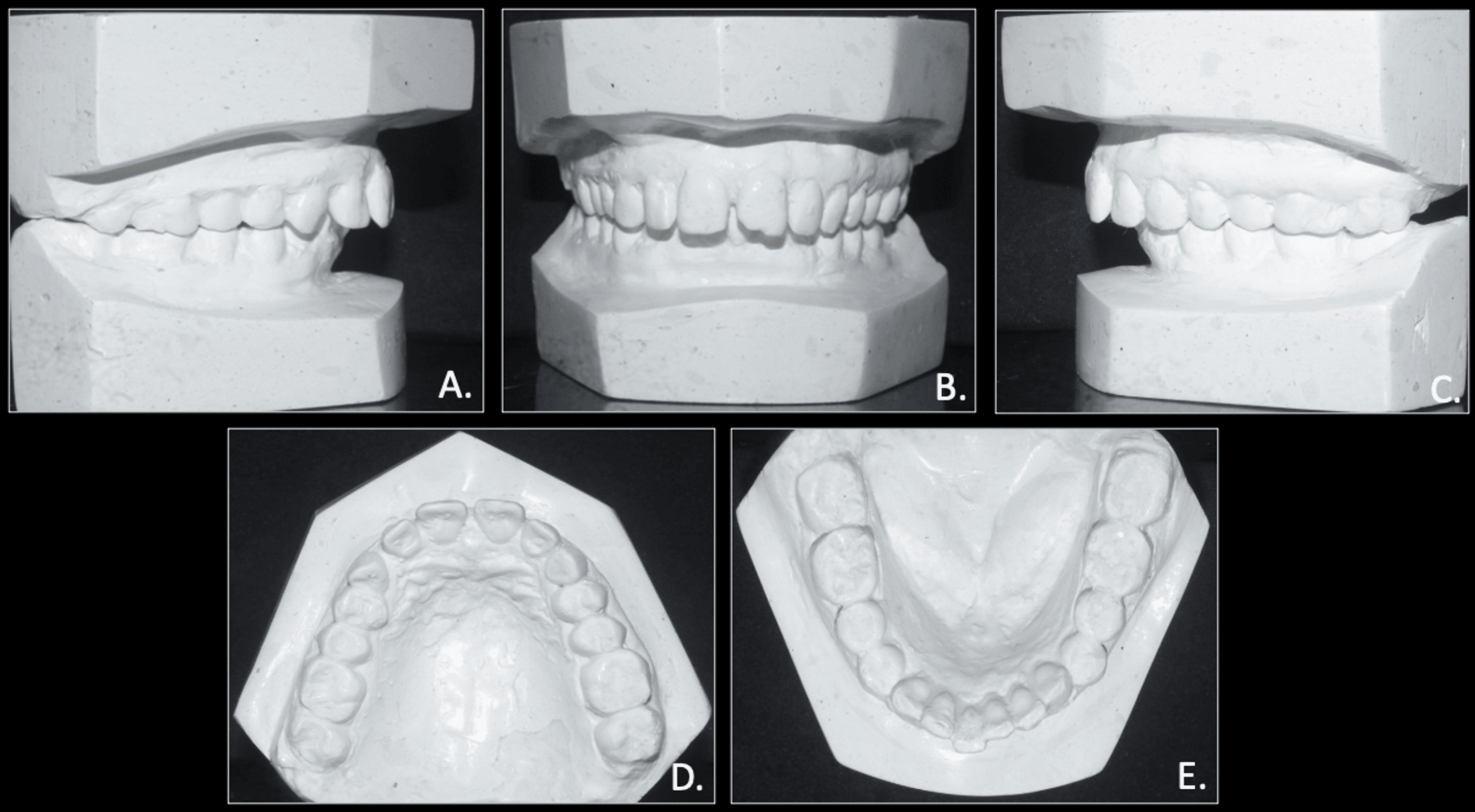
Pre-treatment study models A: right occlusion; B: anterior occlusion; C: left occlusion; D: maxillary model; E: mandibular model

The model analysis of the pre-treatment study models showed spacing of 4.5 mm in the upper arch and 3 mm crowding in the lower arch. The overjet and overbite were calculated to 7.0 mm each. The Bolton tooth-ratio analysis showed larger mandibular teeth than maxillary, with an overall ratio of 91.6% and an anterior ratio of 78.7%, and the Careys analysis shows tooth material excess in the lower arch (Figure 4).

Based on these observations, a 50-year-old female patient was diagnosed with Angles Class II division 1 malocclusion on an underlying Class II skeletal base, with increased overjet and deep bite having a hypodivergent profile.

Treatment objectives

The desired outcomes of the treatment included ideal axial inclination and stable buccal occlusion, ideal overbite and overjet, establishing Class I molar and canine relations, addressing functional problems and path of closure, addressing hypodivergent facial patterns, and establishing an ideal facial profile due to the patient's refusal of surgery. 

Treatment alternative

Initially, orthognathic surgery with the extraction of the lower premolars was proposed to address the skeletal imbalance and facial profile. A non-surgical alternative involving four premolar extractions followed by chin augmentation was also presented. However, the patient declined all surgical interventions and tooth extractions while expressing a desire to improve crowding, spacing, and facial aesthetics. Consequently, treatment options without these procedures were explored.

The treatment plan involved maxillary full arch distalization by orthodontic bone screws to correct the skeletal imbalance and improve the facial profile by opening the mandibular plane angle associated with hypodivergent growth pattern. This approach was facilitated by the absence of both upper third molars. In mandibular arch, to flatten the occlusal plane, the treatment focused on intruding the anterior teeth and extrusion of posterior teeth. Relative intrusion was planned with the establishment of a proper incisor and molar relationship. 

Treatment progress

**Figure 5:**
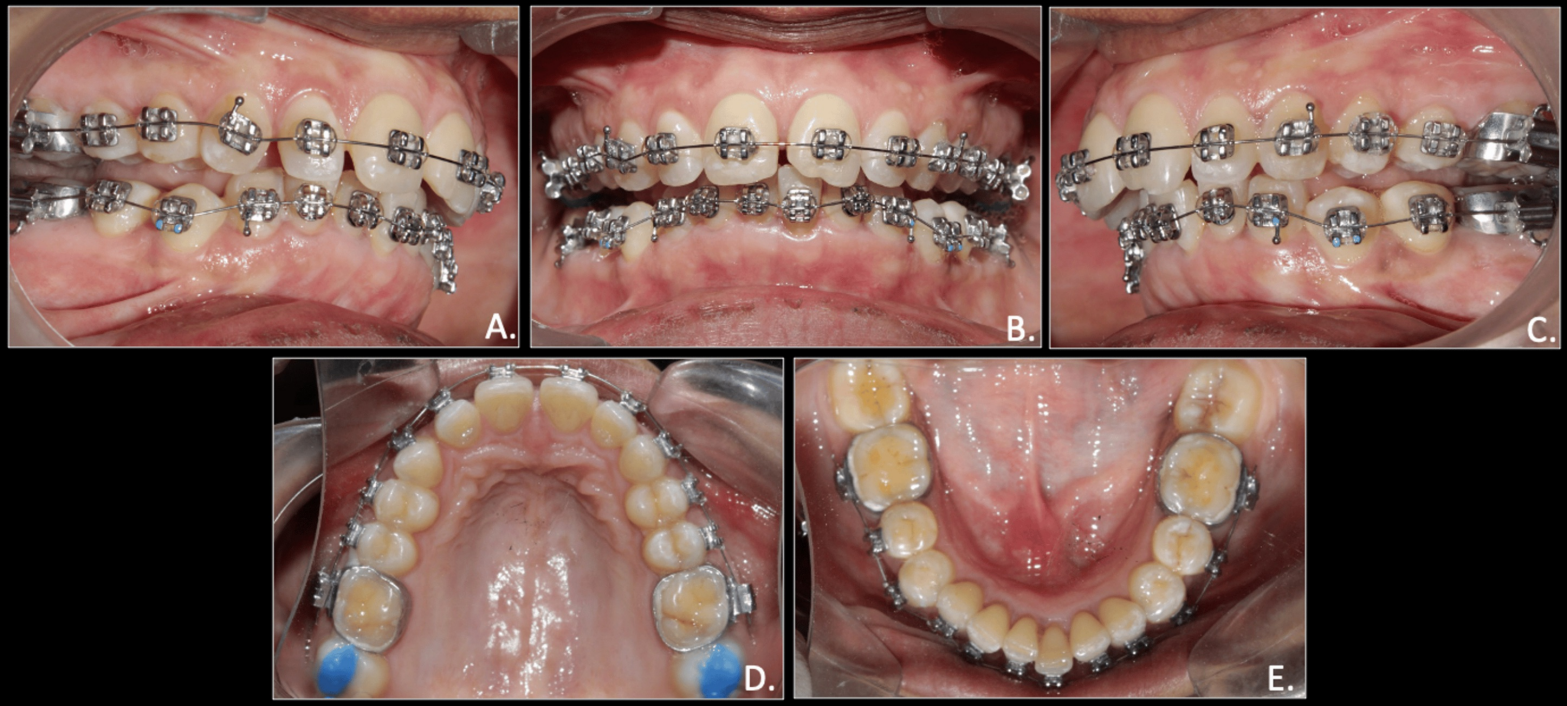
Bonding of upper and lower arches, initial levelling and alignment using 0.022 MBT brackets and 0.012 NiTi wire A: right occlusion; B: anterior in occlusion; C: left occlusion; D: maxillary arch; E: mandibular arch MBT system: McLaughlin, Bennett, and Trevisi system; NiTi wire: Nickel Titanium wire

The 0.022 MBT brackets (McLaughlin, Bennett, and Trevisi system) were bonded and molar banding in the upper and lower arches. Leveling and alignment started with 0.012NiTi (Nickel Titanium) archwires and progressed sequentially to stainless steel (SS) archwires (0.014NiTi, 0.016NiTi, 0.018ss, 17x25ss, 19x25ss) (Figure 5).

**Figure 6:**
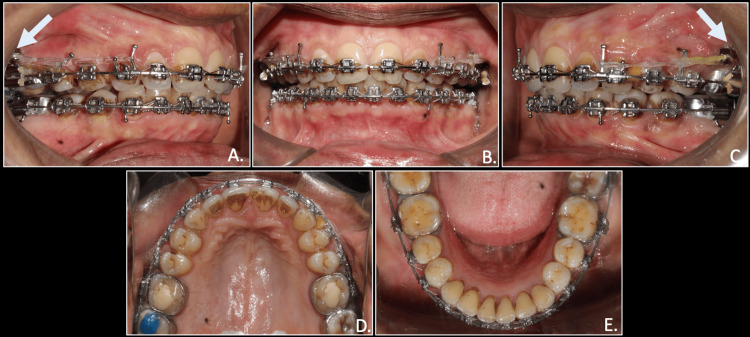
Placement of IZC bone screws with E-chain from crimpable hooks to bone screws for distalization of maxillary arch A & C: (white arrows); A: right occlusion; B: anterior in occlusion; C: left occlusion; D: maxillary arch; E: mandibular arch IZC: infra zygomatic crest; E-chain: elastic chain

Leveling and alignment were achieved, accompanied by space closure in the maxilla and crowding correction in the mandible within a six-month treatment period. Following this, the molar and canine relations became end-on (half-cusp). Once we had achieved leveling and alignment, stainless steel fracture-resistant infra-zygomatic crest screws (IZC) (Bioray, Orthosystems, India) of 12 mm length and 2 mm diameter were then placed at the zygomatic buttress level, superior to both maxillary first molars, to initiate distalization on rigid stainless steel wires. Distalization was initiated with an elastic traction force of 200 g on each side from long crimpable hooks attached in distal to maxillary lateral incisors and between maxillary premolars. To prevent any rotation of the maxillary occlusal plane, a compensatory curve of spee was given in the upper archwire (Figure 6).

**Figure 7:**
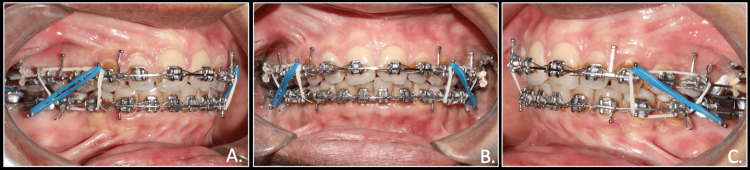
Finishing phase- final settling of dentition after distalization in Class 1 molar and canine relation using settling elastics A: right occlusion; B: anterior in occlusion; C: left occlusion

In the lower arch, a reverse curve of spee (RCS) was given to achieve bite opening and occlusal plane flattening by relative intrusion of mandibular anterior teeth and extrusion of mandibular posterior teeth. Maxillary distalization was performed until a Class I canine and molar relationship was attained, following which the distalization effect was maintained. The final stages of treatment involved achieving optimal occlusal harmony through the use of settling elastics (Figure 7). 

Treatment results

Maxillary arch space closure and total distalization solved her complaint of forwardly placed upper teeth, while clockwise rotation of mandibular plane angle improved hypodivergent pattern to average facial pattern and bite opening due to flattening of curve of spee in lower arch with mild proclination of lower incisors resolved her additional concern of backwardly placed lower jaw. 

**Figure 8:**
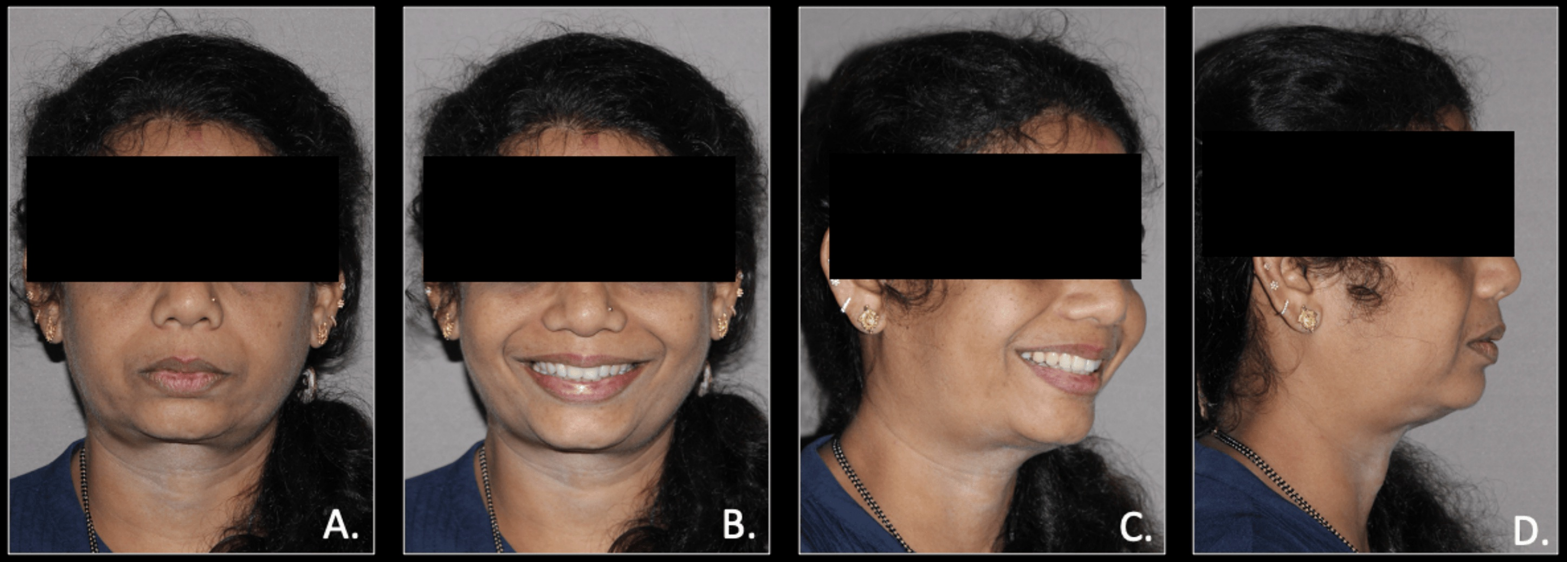
Post-treatment extra-oral photographs A: frontal view; B: frontal smiling view; C: oblique smiling view; D: profile view

**Figure 9:**
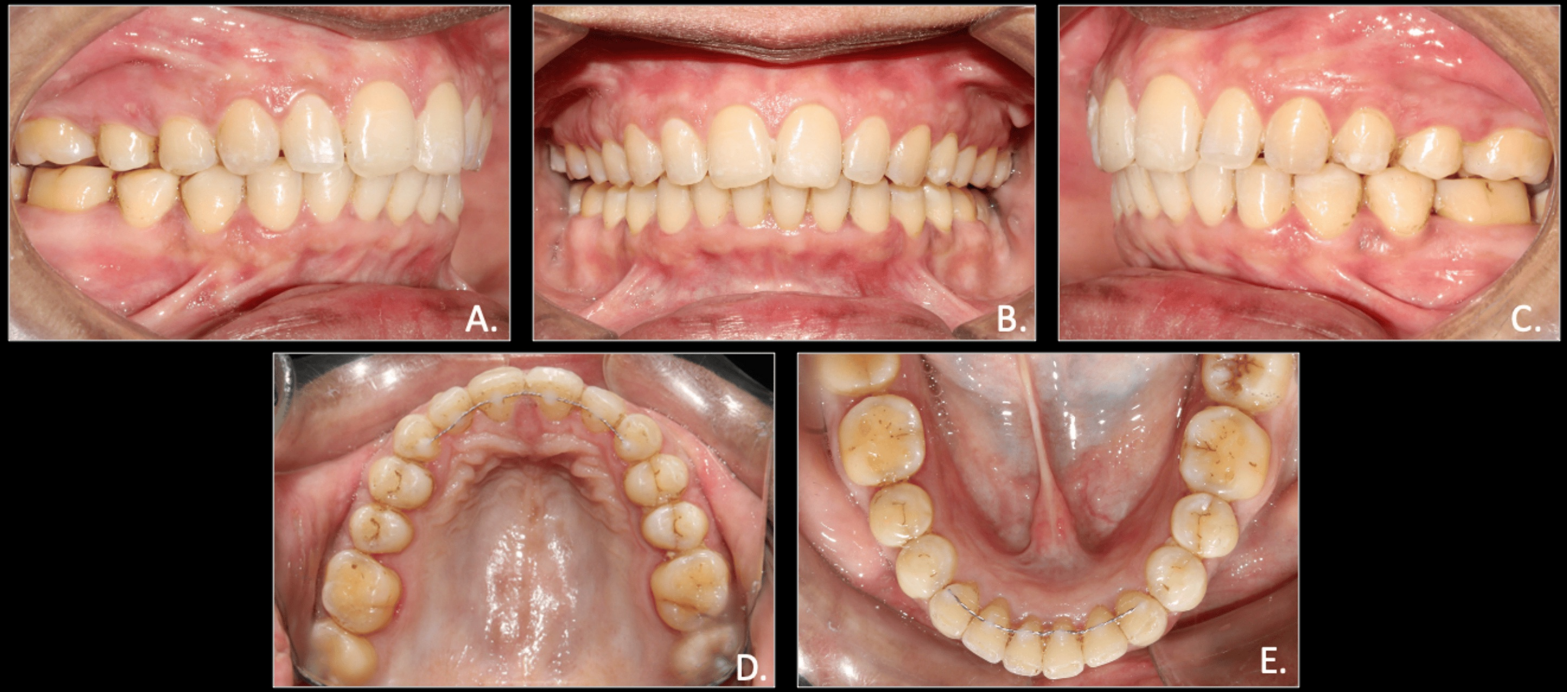
Post-treatment intra-oral photographs A: right occlusion; B: anterior in occlusion; C: left occlusion; D: maxillary arch; E: mandibular arch

Post-treatment, extraoral photographs revealed an improved facial profile with an average facial pattern, an orthognathic facial profile, posterior divergence, reduced lip incompetency, and balanced lip position. Intraorally, the stable and balanced occlusion was achieved with a Class 1 canine and molar relation (Figures 8, 9). 

**Figure 10:**
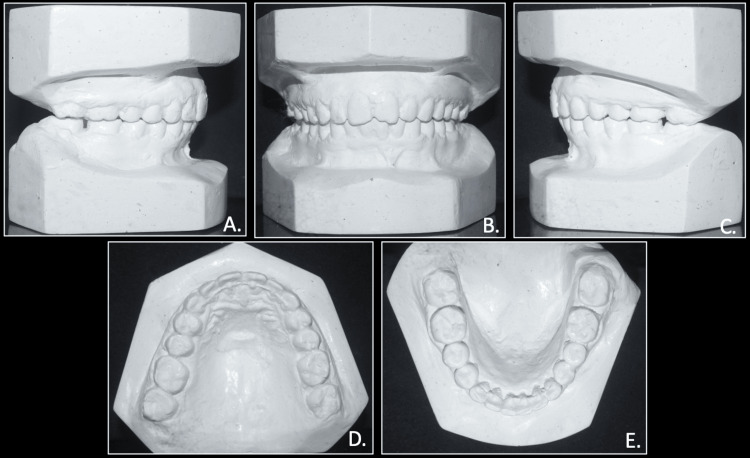
Post-treatment study models A: right occlusion; B: anterior occlusion; C: left occlusion; D: maxillary model; E: mandibular model

The patient achieved the desired overbite and overjet at the end of treatment (Figure 10).

**Figure 11:**
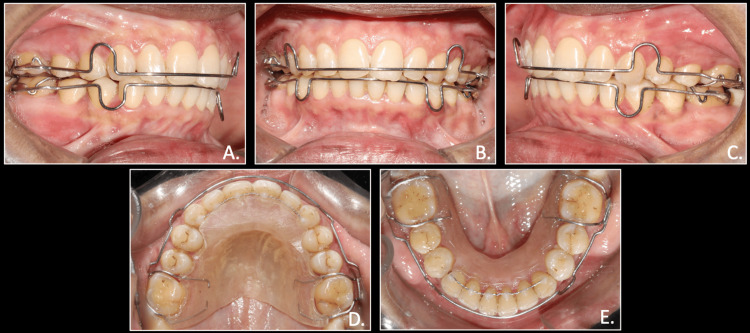
Intra-oral photographs with modified Hawley retainer A: right occlusion; B: anterior in occlusion; C: left occlusion; D: maxillary arch; E: mandibular arch

To achieve post-treatment stability, the treatment results were maintained by fixed lingual retainer and modified Hawley’s retainer (Figure 11).

**Figure 12:**
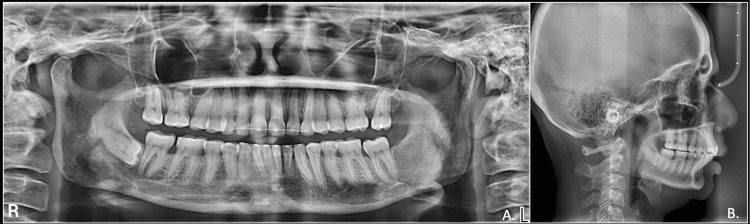
Post-treatment radiographs A: OPG; B: lateral cephalogram OPG: orthopantomogram

**Figure 13:**
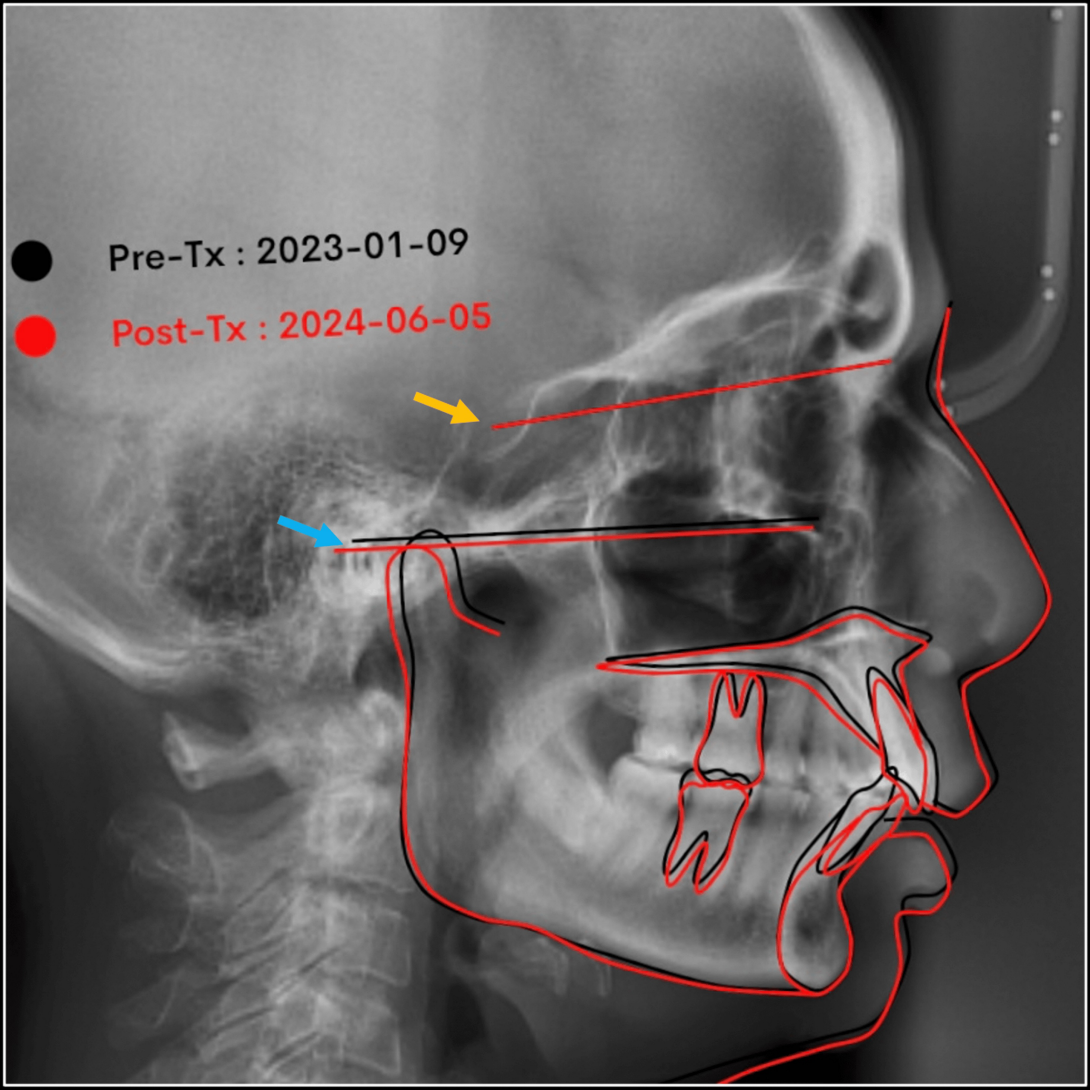
Superimposition of pre-treatment (black line) and post-treatment (red line) lateral cephalograms with reference planes at cranial base (NSL) (yellow arrow) and FH plane (blue arrow) NSL: Nasion-Sella line; FH plane: Frankfort Horizontal plane

The comparison of pre- and post-treatment cephalometric analysis exhibited improved dento-skeletal changes, as anticipated in adult camouflage orthodontics. The maxillary incisor and molars were intruded and distalized by 3.0 mm from the baseline position. In the mandible, the anterior teeth were moved 1.5 mm labially. The patient's facial appearance was enhanced by a reduction in the prominence of the upper lip (2.0 mm) and achieving an ideal lower lip (0 mm) relation with the esthetic line (Figures 12, 13, Table 1). 

**Table 1:** Pre and post-treatment cephalometric values MX: maxilla; MAN: mandible; UI: upper incisors; LI: lower incisors; U. lip: upper lip; L. lip: lower lip; E-line: esthetic line; FH: Frankfort horizontal plane; SN: sella nasion plane; A: point A in maxilla; B: point B in mandible; LAFH: lower anterior facial height; A Pog: point A to pogonion; A vert: point A vertical; S-line: Stiener´s line; N: nasion; FMA: Frankfort-mandibular plane angle

Parameters	Norm	Pre-treatment	Post-treatment
Maxilla			
SNA	81.8sd 3.7	83	80
Nasion perpendicular To A (mm)	-1.2 sd 2.7	-1.5	-1
Effective maxillary length (mm)	93.6 sd 3.2	86*	87
Mandible			
SNB	79.2 sd 2.3	75	78
Nasion perpendicular to Pog (mm)	-3.5 sd 5.3	-11	-9
Effective mandibular length (mm)	120.6 sd 4.5	105*(107-110)	107
MX-MN			
WITS AO/BO (mm)	0 to -1	4.5	2
ANB	2.3 sd 1.1	5	4
Angle of convexity	-8.5 to 10	10	9
Mx-Md diff (mm)	28 sd 3.2	19	20
Vertical			
FMA	24.2 sd 3	22	24
SN-GoGn	32	28	27
Y-Axis	53 to 66	59	62
Jarabaks ratio (%)	62 to 65	68	66
LAFH (mm)	67 - 69	65	69
Saddle angle	123 Sd 5	129	126
Articular angle	143 Sd 6	150	149
Gonial angle	128 Sd 7	116	114
(U)	52 to 55	49	50
(L)	70 to 75	67	64
FH-SN	7	6	5
Base plane angle	25	23	26
Inclination angle	85	80	86
Cant of occ	9	9	8
Maxillary incisors			
UI TO NA (deg/mm)	22/4	24/5	21/4
UI TO A vert	4.2 sd 1.3	7	2
UI TO A POG	-1 to 5	9	6
UI TO FH	107	109	105
UI TO SN	102	103	100
Mandıbular ıncısors			
LI TO NB (deg/mm)	25/4	28/5	30/6
LI TO mandibular plane	90	102	103
LI TO occlusal plane	14.5	28	30
LI TO A-Pog (mm)	1.3 sd 0.7	3	4
MX-MAN			
Interincisal angle	135	118	115
Overjet (mm)	2 mm	7	2
Vertical plane			
Overbite (mm)	15-20%	7/100%	2
Curve of spee (mm)	0 to 1.5 mm	3.5	1
Soft tissue values			
Nasolabial angle	90-110	98	89
L Lip to E-Line(mm)	0-2mm	-1	2
L Lip to S-Line(mm)	0-2mm	-2	0
U Lip to S-Line(mm)	0-2mm	5	0

## Discussion

Camouflage treatment for skeletal Class II malocclusion typically involves tooth extraction to address crowding, dental protrusion, and occlusal relation. Extraoral appliances are often used to distalize upper molars; their effectiveness is dependent on patient compliance. Moreover, these appliances often lead to forward movement of the anterior teeth. To overcome these limitations, orthodontic mini-implants have been employed; in this case, IZC bone screws were placed to manage skeletal Class II as a reliable anchor to achieve controlled molar movement [13,14].

**Figure 14:**
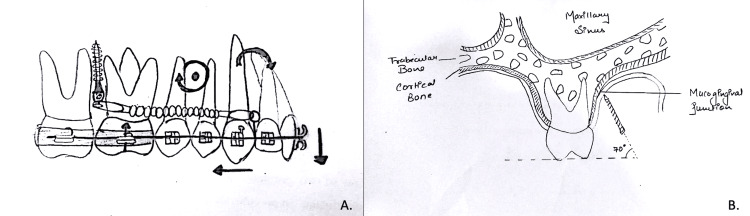
A: Effect of IZC bone screws on maxillary arch; B: IZC bone screws angulation of 70 degrees for distalization IZC: infra zygomatic crest Image credits: Dr. Dhruv Ahuja

To avoid tooth extraction in adult patients in Class II malocclusion, distalization of the upper molars was crucial for successful treatment. Effective distalization using bone screws necessitates careful consideration of biological and mechanical factors, as well as treatment stability. Biological considerations for distalization treatment should include sufficient space in the maxillary tuberosity for distal tooth movement in the upper arch, the inherent limitations of camouflage treatment, and potential interference between bone screws and adjacent tooth roots during full arch distalization [12,15]. The absence of maxillary third molars in this patient provided adequate space in the tuberosity region posterior to the upper molars for tooth movement. This allowed the upper posterior teeth to be repositioned within the existing bone without significant expansion or forward displacement of the anterior teeth, the factors that could compromise gingival health in non-extraction cases. The use of bone screws also helped to minimize unwanted tooth movement, preventing excessive forward tilting of the anterior teeth. To avoid interference with tooth movement, the IZC bone screws were positioned upward to create sufficient space between the implant tip and the root ends. As illustrated in Figure 14, IZC were placed above and between the maxillary first and second molars to achieve overall distalization of the maxillary arch.

Biomechanical factors influencing distalization in the transverse, sagittal and vertical planes were crucial for distalization treatment in this case. In the transverse dimension, the distalizing force exerted by the IZC on the archwire induced buccal crown torque and expansion in the arch due to the wire-bracket interface [15]. To counteract this, palatal crown torque and toe-in bends were incorporated into the archwire design. Despite these measures, mild buccal tipping and intermolar expansion were observed post-distalization. Round stainless steel maxillary archwire with intermaxillary elastics was employed during the finishing phase to address these issues. 

In the sagittal and vertical planes, the posteriorly directed upward force induced lingual inclination of maxillary anterior teeth and occlusal plane canting. The maxillary centre of resistance is approximated at the root tip of the maxillary first premolars. While the maxillary dentition's centre of resistance remains undefined, it is estimated based on individual tooth centres. Vertically, the centre of resistance aligns with individual tooth centres, and sagittally, it is positioned between the first and second premolars for entire maxillary dentition [15,16]. Consequently, the distalizing force from the IZC to the lateral incisor-canine crimpable hook induced clockwise maxillary rotation which resulted in anterior tooth lingual inclination and occlusal plane cant alteration. Given the U1-SN angle within normal limits (103°) and the planned occlusal plane stability, a 15° compensating curve was incorporated into the archwire to counteract clockwise maxillary rotation. Regular lateral cephalometric evaluations monitored these parameters.

Treatment stability for maxillary distalization in skeletal Class II malocclusion hinges on IZC success rate, periodontal remodelling, and arch stability. While IZC success rates approach 90%, loosening remains a possibility [17]. Pre-treatment discussions of alternatives like headgear, additional stripping, and arch expansion were crucial. The patient was informed of these contingencies and consented to the treatment plan [14,16]. Periodontal remodelling is a critical consideration during molar distalization. As the maxillary second molars were distalized into the tuberosity region abundant in attached gingiva, the upward and backward movement of molars to maintain vertical dimension induced pseudo-pockets of greater than 3mm, particularly distally. Post-distalization gingival swelling around the maxillary second molars was resolved following periodontal therapy involving irrigation and oral prophylaxis [15,16].

Treatment outcomes in adult patients are dependent upon a confluence of factors. In this specific case, a combination of biomechanical principles and the patient's resolute preference for non-surgical orthodontics facilitated the successful application of maxillary en-masse distalization, with mandibular incisor intrusion, mandibular posterior extrusion, and occlusal plane flattening. This comprehensive approach culminated in appreciable enhancements to the patient's dento-skeletal and soft tissue profile.

## Conclusions

In adult patients exhibiting skeletal class II malocclusion, bone screws facilitate distalization, correction of posterior occlusal discrepancies, reduction of excessive overjet, and improvement of deep bite associated with hypodivergent facial patterns. By meticulously considering biological and biomechanical factors, as well as post-treatment stability, predictable and enduring treatment outcomes can be achieved. The use of bone screws in orthodontic treatment can provide a less invasive alternative to orthognathic surgery, often yielding similar outcomes.
